# The Adaptation and Retention of Artificial Dentures

**Published:** 1898-03

**Authors:** A. O. Hunt

**Affiliations:** Chicago, Ill.


ARTICLE VI.

### THE ADAPTATION AND RETENTION OF ARTIFICIAL DENTURES.

BY A. O. HUNT, D. D. S., CHICAGO, ILL.

While this is an old subject, yet it is new and will continue to be so until there is some better agreement as to the principles involved in the o[illegible]eration.

These principles will not be well established unless they are based upon actual conditions present in mouths. They must also be broad enough to apply all the variations that present themselves.

Atmospheric pressure is one of the forces claimed as being essential in retaining the dentures, the pressure being produced by a central chamber in which a vacuum is produced. The force exerted is estimated to be from a few ounces to fifteen pounds to the square inch. This claim is entirely faulty. A vacuum cannot be produced in this way, and if it could, would only be of very short duration. Again, if it were possible to get the benefit of such force, it would be better to have it extend over the entire surface of the denture than to confine it to a small central area. Still further, it cannot be applied to lower dentures successfully.

Capillary attraction or adhesion is another force mentioned as all important in the retention of the denture. As whatever force of this character that might be utilized is based upon the laws of cohesion and adhesion, the limit of its operation is so small that it would be insensible in an artificial denture. If, however, the force was greater, it would be better that it should apply to the whole surface than to a part of it.

The conditions presented in the upper jaw are peculiar many times. The idea that a mouth that is flat is more difficult than a deeper one to secure good results should not be entertained. In general, in from six months to a year after the loss of the teeth, there are not so many difficulties in the way of good results. After a denture has been worn for a long time with only a partial antagonism with a few opposing teeth, the difficulties are multiplied. So also when the mouth is edentulous and no denture has been worn for a long time with only a partial antagonism with a few opposing teeth, the difficulties are multiplied. So also when the mouth is edentulous and no denture has been worn.

When the best model obtainable is at hand, a careful digital examination should first be made of the surface of the mouth, to locate and define the depth and form of the soft spaces within the area to be covered by the plate. These spaces and their form and depth should be outlined on the model, the depth indicated by figures, as it varies greatly in corresponding locations in the same mouth.

The *model* should then be scraped away in the various places to the proper depth so that the denture will bear evenly over its whole surface, keeping in mind that the central or palatal portion will not change materially, while the alveolar ridge is constantly changed by resorption.

For the upper jaw there are five localities where the denture will find a firm and reasonably permanent rest without undue pressure on the parts.

The palatal surface, two on the labial surface immediately over the region occupied by the cuspids, and two over the malar processes extending to and above the malar processes, extending to and above the maxiliary tuberosities.

For the lower jaw there are four localities for a firm rest for the plate, two on the labial in the region of the cuspids and two on the posterior buccal margins between the summit of the alveolar ridge and the attachment of the buccinators to the inferior maxillary. The greatest interference with a denture being retained in its place, as they are usually constructed, consists in the action of the muscles upon the margins of the plate to push it either up or down as the case may be, or oftentimes the posterior margin of the upper one extends upon the soft palate and is thrown down by its movement; or in the lower denture the posterior ends of the plate are so long that in opening the mouth the stretching of the tissues in front of the ramus move it forward.

While all the muscles may throw a deuture out of position, yet by a proper form of the margins, some of them may be utilized as the most important force for retention; notably, the buccinators and the orbicularis oris.

A careful examination of the attachments of these muscles should be made, and in shaping the margins their movement should be opposed to each other in such a way that they will be compressed upon the denture and hold it firmly.

If improperly made, patients never get the use of dentures until they learn to hold them in place by the buccinators, assisted by the tongue and the orbicularis oris.

With an even pressure over the entire surface of the plate in contact with the mouth, a free movement of the anterior frænum and the anguli oris muscles, the anterior border of the buccinator, the compression of the buccinator at the posterior borders and the compression of the orbicularis uris at the anterior portions, a free movement of the lingual and sublingual muscles on the interior surface of the lower denture, with plenty of room for the tongue, accompanied with a stable pressure on the hard or permanent parts of the mouth, will produce a perfect adaptation and retention of an artificial denture.—*Dental Review.*

